# Biosensor Applications in the Field of Antibiotic Research—A Review of Recent Developments

**DOI:** 10.3390/s111009450

**Published:** 2011-10-03

**Authors:** Katrin Reder-Christ, Gerd Bendas

**Affiliations:** Pharmaceutical Chemistry, Rheinische Friedrich-Wilhelms-Universität Bonn, An der Immenburg 4, D-53121 Bonn, Germany; E-Mail: gbendas@uni-bonn.de

**Keywords:** antibacterials, antibiotics, biosensor, bacterial resistance

## Abstract

Antibacterials are among of the most important medications used in health care. However, their efficacy is increasingly impeded by a tremendous and globally spread bacterial resistance phenomenon. This bacterial resistance is accelerated by inadequate application of antibacterial drugs in humans, the widespread veterinary use of antibacterials, and antibacterial occurrence in the environment and food. Further, there is a lack of development of innovative novel drugs. Therefore, the search for novel antibacterials has to be intensified and the spread of antibacterials in the environment has to be restricted. Due to the fundamental progress in biosensor development and promising applications in the antibiotic field, this review gives for the first time an overview on the use and prospects of biosensor applications in that area. A number of reports have applied biosensors of different design and techniques to search for antibacterials in environmental and foodstuff matrices. These studies are discussed with respect to the analytical values and compared to conventional techniques. Furthermore, biosensor applications to elucidate the mode of action of antimicrobial drugs *in vitro* have been described. These studies were critically introduced referring to the informational value of those simulations. In summary, biosensors will be illustrated as an innovative and promising, although not yet comprehensively applied, technique in the antibacterial field.

## 1. Introduction—Bacterial Resistance, Prospects of Antibacterial Development and the Use of Biosensors in This Field

**Figure 1.**
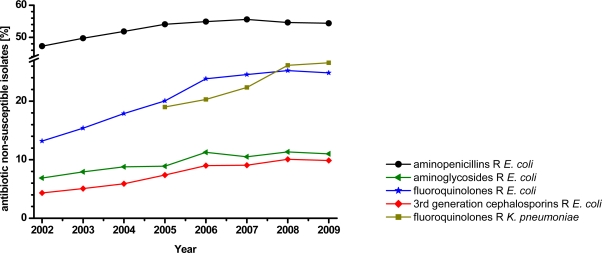
Progress of selected resistant bacterial strains in Central and Southern Europe between 2002 and 2009. The amount of resistant isolates increased slowly but constantly over the years.

Antimicrobial drugs (antibiotics either as compounds of microbiological origin, their partial synthetic derivatives or chemically synthesized compounds—collectively termed “antibacterials” in this review), more than any other class of drugs, have accounted for an increased life expectancy in humans. However, the efficacy of antibacterials is increasingly impeded by a tremendous and globally spread occurrence of bacterial resistance against these treatments—a phenomenon that arose with the discovery of antibacterial drugs and remains an increasing problem. Nowadays, resistance affects virtually all major bacterial pathogens and all types of epidemiological settings. As an example of the situation worldwide, Figure 1 shows the progress of selected resistant bacterial strains in Central and Southern Europe between 2002 and 2009 (data collected by the European Antimicrobial Resistance Surveillance System, EARSS [1]).

During continuous exposure to antibacterials, sequential chromosomal mutations can occur, leading to the appearance of resistance mechanisms step by step [2,3]. Several factors contribute to the occurrence of bacterial resistance: (i) the inappropriate use/misuse of antibacterials in humans; (ii) the veterinary use of antibacterials in pets, farm animals and animals raised in aquaculture [4]; and (iii) the increased occurrence of antibacterials or their metabolites contaminating the environment, mainly resulting from the latter applications.

**Figure 2.**
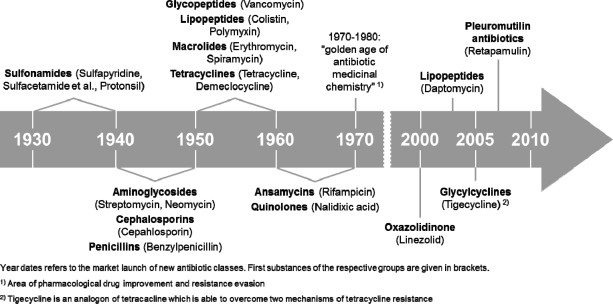
The timeline of antibacterial development modified from Wright [7]. Antibacterial development started in the 1930s with the sulfonamides, followed by a period of 40 years with successful antibacterial discovery. After a long period in which no real novel antibacterials were discovered, only a few new antibacterial classes were identified in the current millennium.

To ensure the efficiency of antibacterial treatments in the near future, the further development of bacterial resistance has to be stopped, *i.e*., by reducing the contamination of the environment with antibiotics. Furthermore, it is equally important to develop novel antibacterials or to identify natural compounds that can overcome the existing resistance mechanisms or attack novel target structures. The development of new antibacterial substances, however, is still a problem. For example, despite the fact that lower respiratory infections were the third leading cause of death worldwide in 2008 and the leading cause in low-income countries [5], many pharmaceutical companies have withdrawn completely from antibacterial drug discovery because the development of new drugs is increasingly expensive before getting approval. Once new entities reach the market, it is difficult to recoup the costs of development, which is especially true for antibacterials due to their mainly short time application intervals [6]. This is reflected in the time scale of antibacterial development (Figure 2).

Until the early 1980s different antibacterial classes were identified. Then, until the end of the 20th century, there was no further development of real novel antibacterials, which strengthens the present efforts to find novel modes of action or to attack hitherto unknown target structures [7]. In recent years three novel antibacterials (the lipopeptide daptomycin [8], the glycylcycline tigecycline [9] and the pleuromutilin antibacterial retapamulin [10]) have been approved. All other approved antibacterials are more or less derivatives of known ones. Moreover, there are presently only five real novel antibacterial agents in clinical development, necessitating the search for new sources, such as marine natural products or for new bacterial targets in plasma and outer membrane, cell wall, ribosomes, nucleoid, or plasmids.

**Figure 3.**
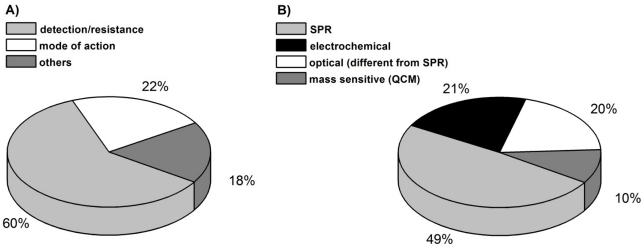
Biosensor applications in the antibacterial field are classified regarding to (**A**) their experimental approaches, and (**B**) the detection principles. (A) Nearly 60% of the biosensor applications contribute to detection or quantification of antibacterials in the environment as a basis for interference with the increasing bacterial resistance; (B) Nearly half of the detection principles are based on surface plasmon resonance technique.

The following review will highlight biosensors as useful tools in the antibacterial research field, their usefulness to fight against the spread of bacterial resistance, and the development of new antibacterial compounds. The number of biosensor approaches in this area has increased during the last five years, dominated by studies for the detection of antibacterials in the environment [Figure 3(A)]. In the first part of this review a number of studies, which have applied different biosensor methods to detect antibacterials in various matrices with the intention to stem resistance developments, will be interpreted and evaluated with respect to the technical merits and prospects.

The second part focuses on the use of biosensors in mode of action studies of novel antibacterials. Although there are fewer studies for the latter application [Figure 3(A)], the prospects of biosensors to participate in drug development are certain.

It must be noted that this review can only give an instantaneous view in the current situation of biosensor application in the antibacterial field. The given information (especially that in Figure 3) is based on the availability of published data [11]. However, the authors cannot exclude the existence of much more, as yet unpublished applications of biosensors mainly in the field of industrial antibacterial development. As mentioned before, the development of new antibacterials before approval is a slow and strictly regulated process. One can assume that the number of applications of biosensor techniques in the search for new antibacterials is higher than demonstrated by the authors. Nevertheless, this review and the data therein are restricted to already published data and, in the case of the antibacterial timeline (Figure 2), to approved drugs.

## 2. Biosensor Application to Detect Antibacterials in Environment and Food as Contribution to Prevent Bacterial Resistance

### 2.1. General

The growing incidence of resistant bacteria due to inadequate medical or veterinary treatment regimens is greatly intensified by the subsequent appearance of antibacterials in the environment. Environmental accumulation and pollution is not only a problem of antibacterials, but also relevant for different biologically active compounds in general (drugs, drug metabolites or endocrine disruptors). Due to bacterial adaptation and resistance formation, antibacterials merit special attention. The analysis of drinking water is of special interest in this context given the different pathways and possibilities to place potential pollutants into aquatic circulation. However, there are no standardized analytical methods for aquatic pollutants. Nowadays the main analytical tools are liquid and gas chromatographic techniques, mostly combined with mass spectrometric analysis. For detailed information on current analytical techniques for the detection of aquatic pollutants, the reader is referred to reviews in this field ([12–18] and the references therein). The analysis of antibacterials, for example, in environmental waters is usually performed by HPLC-MS or HPLC-MS/MS. Other methods, such as UV or fluorescence spectroscopy or electrochemical detection are of minor importance due to their lower sensitivity.

Since antibiotics are given to animals for therapy and prophylaxis as well as to increase growth and feed efficiencies [4], the proof and control of antibiotics in animals and foodstuffs of animal origin is essential. In the food industry milk is one of the most heavily regulated products. Due to their lipophilic properties, antibacterials can easily accumulate in higher amounts in milk. The widespread usage of milk makes it necessary to control threshold levels of antibacterials in milk. It is essential to detect the antibacterials prior to a contamination of the food chain event, e.g., at the farm. Therefore analytical methods for simple and speedy detection are necessary.

It is well known that the above mentioned “conventional” analytical methods are elaborative with respect to sample preparation (pre-treatment, clean-up and concentration), costs and time consumption and often require personal expertise and skills. The ongoing development of biosensors opens a promising new way to enable the use of fast, simple and sensitive techniques to detect environmental pollution by antibacterials or their appearance in food products.

The application of biosensors as tools to detect pathogens is beyond the scope of this review. Besides the analysis of pathogens in the human organism, such as analysis of *Chlamydia trachomatis* in urine using optical biosensors [19] or causative organisms of dengue fever using quartz crystal microbalance (QCM) [20], the detection of e.g., pathogen-infected foodstuffs is essential. Examples of biosensor applications are the detection of aflatoxin in milk samples [21], the detection of *E. coli* O157:H7 in food samples [22] or the use of a cell based biosensor technique to detect various numbers of pathogens and toxins [23].

Biosensors as detection tools for antibacterials clearly differ in the sensor system, the principle of sample recognition as well as in the type of matrix. The different recognition and detection principles will be pointed out below.

### 2.2. Recognition and Detection Principles for the Biosensor Based Detection of Antibiotics

In general, there are two main principles for the recognition of antibacterials by biosensor systems. The first one comprises the widespread use of immobilized aptamers as recognition elements (so called aptasensors) [24–27]. RNA and DNA aptamers are oligonucleic acids that bind the analyte of interest by their 3D-structure via ionic interaction, van-der-Waals-forces or hydrogen bonds leading to detectable signals. Their sensitivity is comparable to that of antibodies. Furthermore they can be chemically synthesized, possess a high thermal stability and are easy to modify and to immobilize. The second principle of antibacterial recognition for biosensing is given by antibody-mediated binding processes. Those immunosensors have been widely used for antibacterial detection [28–34]. It is either possible to immobilize antibiotic-specific antibodies at the sensor surface to directly detect the antibiotic binding, or to invert the assay and detect the binding of antibody-spiked samples onto immobilized antibiotics in terms of a competitive assay.

Beside aptamers and immunoassay-based recognition, other principles have to be mentioned, e.g., the use of enzymes or functionalized gold nanoparticles or the application of whole bacterial cells as recognition elements.

Referring to enzyme-coupled principles, a number of studies have reported on the immobilization of β-lactamase for the *in vivo* detection of penicillins [35–37]. Hydrolysis of the penicillins led to a decrease in the pH value, which was amperometrically detected by Chen *et al.* [37], amongst others in milk samples.

A very interesting recognition approach is the use of functionalized gold nanoparticles. Frasconi *et al.* [38] protected gold nanoparticles with thioaniline (as electropolymerizable unit), mercaptophenylboronic acid (as ligand for antibiotics) and mercaptoethanesulfonic acid (for nanoparticle stabilization). The polymerization of these functionalized nanoparticles on a gold surface was followed by SPR in the presence of aminoglycosides (neomycin, kanamycin, streptomycin) and used as a sensor for antibacterial detection in milk samples. SPR signals are amplified by the use of nanoparticles and thus, the sensor sensitivity is increased.

In contrast to the above mentioned technical biosensor devices, a number of studies also referred to “biosensing” antibacterials using whole bacterial cells with specific detection and sensing mechanism for certain antibacterials. One example is given by Virolainen *et al.* [39], who introduced an *E. coli* bacterial strain containing a luciferase operon placed under control of a tetracycline response element. Therefore, these bacteria, which can be kept in a freeze-dried form, produce self-bioluminescence following tetracycline recognition. The evaluation can be performed in a plate assay format as an essential prerequisite for a rapid, inexpensive high-throughput screening system. The authors described the detection of different tetracyclines in poultry muscle tissue in the low ng/g range meeting the demands of the maximum residue levels (MRL) of the European Union. A follow-up study, which compared the capacity of the bacterial sensor with microbiological inhibition assays or LC-MS/MS detection of tetracyclines in routine analyses of poultry samples, confirmed the value and applicability of this approach [40].

Despite many different antibacterial recognition principles that can be used for biosensor applications, only a limited number of biosensor techniques were used in the antibiotic field. Figure 2(B) illustrates the most common methods for analyte detection in that area. Some 50% of the biosensors used are based on the SPR technique. SPR can be upgraded by imaging methods (iSPR) to get more details on the antibacterial binding process [32,41].

Optical detectors that are different from SPR account for about one fifth of antibacterial detection. Alongside the already mentioned detection of luminescence after antibiotic binding, fluorescence measurements were also applied [28,35].

Electrochemical methods (21%) are the second most used detection principles beside SPR. They are dominated by voltammetric (cyclic voltammetry/CV or square wave voltammetry/SWV) and amperometric methods. Thereby a reduced Faradaic current is detected after antibiotic binding. The antibacterial layer impedes the electron transfer acting as an electrode isolating layer. Since antibacterials are most often not electrochemically active by themselves, redox tags like hematein, methylene blue or the commonly used Fe^2+^/Fe^3+^ system have to be added. Impedance spectroscopy as an electrochemical detection method was also described and works without redox tags.

In principle there are versatile ways to combine the different recognition and detection principles. However, some combinations are more common in the antibiotic field. For instance, the binding of antibiotics to aptamers is often detected by electrochemical methods (CV and SWV). Zhang *et al.* and Kim *et al.* used DNA aptamers for the detection of tetracycline and oxytetracycline. Zhang *et al.* [24] coupled a tetracycline binding ssDNA-aptamer via EDC/NHS-chemistry on a glassy carbon electrode. Cyclic voltammograms of defined tetracycline concentrations were recorded in the presence of redox-active K_3_[Fe(CN)_6_]. The correlation between increasing tetracycline concentrations and decreasing current peaks (due to an increasing isolation of the electrode) was used for fast detection of unknown tetracycline concentrations in milk. Kim *et al.* [25] immobilized an oxytetracycline binding ssDNA-aptamer with high affinity, while the binding of other tetracyclines was discriminated. Since the aptamers was thiol modified, immobilization could easily be performed occurred via covalent chemistry on a gold electrode. CV and SWV were applied to determine oxytetracycline concentrations. RNA aptamers were applied by Rowe *et al.* [26] to electrochemically detect the concentration of aminoglycoside antibiotics in blood samples in order to prevent overdosage and side effects. Aminoglycoside binding RNA aptamers were immobilized on a gold electrode. To avoid fast degradation during application in blood samples, the aptamers were modified by methylation of all 2′-hydroxyl groups outside the antibiotic binding pocket. As an alternative, more degradation-resistent DNA-aptamers were applied. SWV experiments showed a lower sensitivity of the DNA-aptamer based sensor to detect aminoglycosides in blood samples compared to RNA-based sensor.

Electrochemical detection was also combined with immunosensor based recognition. For example, Ionescu *et al.* [33] immobilized anti-ciprofloxacin antibodies onto a polypyrrole-NHS-film. A positive interaction with ciprofloxacin was detectable by changes in the impedance spectrum. As an alternative assay, ciprofloxacin was immobilized on the polypyrrole-NHS-layer and covered with ciprofloxacin antibodies by Giroud *et al.* [29]. Since ciprofloxacin from a sample solution binds to the antibody with a higher affinity compared to the immobilized derivatives, the resulting removal of the antibodies relates to the sample ciprofloxacin concentration, which can be monitored and even quantified by recording the impedance spectra. This approach corresponds to a competitive immunoassay.

A very common combination is the immunosensor based recognition of antibacterials followed by an SPR based detection–a fact that might be due to the broad spread of both. The competitive immunoassay as biosensor recognition element has, for example, also been applied by Dong *et al.* [30] for the detection of chloramphenicol in pork. Ferguson *et al.* [34] immobilized a streptomycin derivative and analyzed the competitive binding of a mixture of streptomycin and an anti-streptomycin-antibody. Thus, the quantitative analysis of streptomycin and dihydrostreptomycin in honey, milk and meat became possible. Both used SPR as detection method. SPR as the most common detection method was also combined with aptamer recognition. The aminoglycoside neomycin B was also detected with the help of an RNA aptamer by de-los–Santos-Alvarez *et al.* [27].

### 2.3. Biosensor Tools to Detect Antibiotics in Food

After introducing the different recognition and detection principles, this chapter will present biosensors as fast and easy-to-use devices for the detection of antibacterial residues in milk and other food products. These devices exceed traditional time-consuming monitoring methods and are sensitive enough to detect antibiotic concentrations below the maximum residue levels (MRL), which have been defined by the European Union. Due to the EU Council Regulation No 2377/90 the “maximum residue levels” are defined as “the maximum concentration of residue resulting from the use of a veterinary medicinal product which may be accepted by the community to be legally permitted or recognized as acceptable in or on food. It is based on the type and amount of residue considered to be without any toxicological hazard for human health as expressed by the acceptable daily intake [42]”. Here “food” comprises all the various foodstuffs of animal origin, including meat, fish, milk, eggs and honey. MRLs were established for many antibacterials and their adherence has to be controlled by suitable and sensitive analytical techniques.

**Figure 4.**
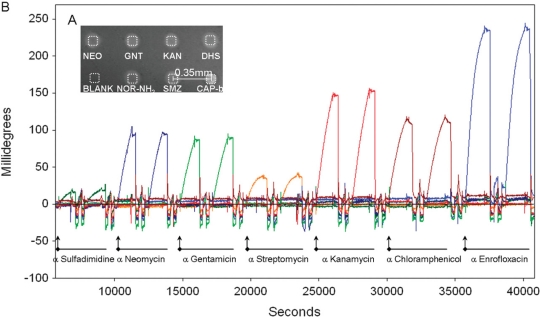
(**A**) SPR image of antibody binding onto different immobilized antibiotics (neomycin/NEO, gentamicin/GNT, kanamycin/KAN, dihydrostreptomycin/DHS, norfloxacin-NH_2_ derivative/NOR-NH_2_, chloramphenicol/CAP, and sulfamethazine/SMZ. **(B)** Sensorgramms display specific antibody recognition with low cross reactivity (reprinted with permission from Rebe Raz, S.; Bremer, M.G.E.G.; Haasnoot, W.; Norde, W. Label-free and multiplex detection of antibiotic residues in milk using imaging surface plasmon resonance-based immunosensor. *Anal. Chem.* **2009**, *81*, 7743–7749. Copyright © 2009, American Chemical Society).

Rebe Raz *et al.* [41] reported on the simultaneous quantification of up to seven antibiotics from different families in buffer or milk by implementing a microarray immunoassay in an imaging surface plasmon resonance platform. On the basis of NHS/EDC chemistry the authors immobilized sulfamethazine, neomycin, gentamicin, streptomycin, kanamycin, chloramphenicol, and entrofloxacin on a SPR sensor with the help of a microspotter. The assays’ sensitivity and specificity were controlled by following the binding of the corresponding antibodies. Although the SPR signals were comparably low, they clearly demonstrated specific antibody binding with low cross reactivity (Figure 4).

The validated sensor was then used to follow the inhibition of the antibody binding in a competitive format by the relevant antibiotics in the test solutions. The mentioned antibiotics in both buffer (HBS) or 10-time diluted milk were quantified in the low ng/mL range (IC_50_ values), whereas a uniform matrix effect of the milk constituents was not evident. The calculation of the limit of detection showed sensitivity in the ppb-range, which was sensitive enough for milk control at the MRL level established in the EU for neomycin, kanamycin, streptomycin, entrofloxacin, and sulfamethazine. This rapid and label-free detection of antibiotics from the aminoglycoside, fluoroquinolone, phenicol, and sulfonamide family opened the perspective for an automated and high-throughput monitoring of antimicrobial drug residues in food.

A comparable approach to detect different antibiotics in milk using an SPR device has recently been reported by Fernandez *et al.* [43]. The focus was the establishment of a compact and portable device with a six channel configuration suitable for on-site applications. Haptenized proteins were immobilized by the EDC/NHS coupling procedure in the different channels and enrofloxacin, sulfapyridine, and chloramphenicol were detected as representatives of the fluoroquinolones, sulfonamides, and phenicols, respectively, by adding the respective polyclonal antibodies to the samples. Matrix effects of milk could simply be reduced by five-time dilution of the milk with buffer. No further pre-processing steps of sample preparation were needed. A very good degree of repeatability of detection on consecutive cycles with coefficients of variation below 5% was reported. The limit of detection of enrofloxacin and sulfapyridine in the low μg/kg range was far below the MRL and for chloramphenicol slightly superior to the MRL. It was considered that the SPR device has identical analytical capacities compared to other commercially available SPR systems and approaches in this field [41,44,45] but additionally offers the advantage of compactness and mobility. With respect to the group-selectivity of the used immunoreagents, a much higher number of antibiotics from the included families appear to be detectable with this analytical approach.

A similar approach of milk analysis, but using an alternative biosensor system has been reported by Adrian *et al.* [46]. An immunosensor on the basis of a wavelength interrogated optical sensing device in a flow chamber system was developed to detect the binding of sulfonamides with immobilized immunoreagents. Sulfapyridin as reference was detectable at a low μg/L level, and milk samples contaminated with different sulfonamides were identified according to the MRLs of the EU. The same technique has been applied to develop a multiplexed biosensor to detect sulfonamides, fluoroquinolones, and tetracyclines in raw milk [47].

Referring to SPR analysis of antibacterials in other food matrices, Huet *et al*. [48] reported on the development of an immunochemical screening method based on SPR for the detection of 13 different fluoroquinolones with special focus on flumequine in egg, fish, and poultry meat. A bi-active antibody for immune recognition was prepared and enables the detection of 13 different fluoroquinolones at levels below their established MRLs after a simple liquid extraction procedure. Comparison with conventional analytical approaches (LC-MS/MS) in this study and a follow-up study including the microbiological assays [49] confirmed similar sensitivity of this biosensor method and the potential as a routine analysis of fluoroquinolones in the mentioned matrices.

In a similar approach, the validation of a Biacore-based system for detection of sulfonamides in porcine, bovine and poultry muscle was reported [50]. The performance characteristics of the system were evaluated with respect to the MRL demands of the EU and found to be sufficient with respect to the detection limits, but also to precision and applicability.

The selected examples justify the conclusion that biosensors are suitable and very promising tools for the search of antibacterials in the environment and appear as an important component to stem the distribution of bacterial resistance. Despite many different techniques that could be used as biosensor detection principles, only a limited number of principles are presently used in the antibiotic field with a dominance of the SPR technique. Other optical as well as electrochemical detection principles also contribute to antibiotic recognition whereas, for example, mass sensitive biosensors are underrepresented in this area. Compared to conventional analytical methods, biosensors can be applied as fast, simple and cost efficient methods that can even be used without further sample preparation. The examples discussed above show that the detection limit is comparable to that of conventional analytical methods and fulfils the criteria threshold values of common regulatory requirements and the MRL rules of the EU.

However, the data does not allow a final conclusion whether biosensors presently have potential as a high throughput screening application. Promising biosensor developments with strict orientation for high throughput applications, such as the kinexa systems, for the detection of pollutants in the aqueous environment as example [51], have not been described in the antibiotic field.

## 3. Biosensor Applications to Elucidate the Molecular Mode of Action

### 3.1. General

In order to fight bacterial resistance it is equally important to develop novel antibacterials and to identify natural compounds that can overcome the existing resistance mechanisms or to attack new target structures. In addition to the previously mentioned control and detection of antibacterials in the environment, biosensors can also add to the search for novel targets and simulated processes of antibacterial activities. The following section will provide a summary of studies where biosensors, mostly SPR and mass sensitive biosensors, were applied to obtain further insights into the mode of action of established or experimental antibacterials or in the search of bioactive natural compounds.

### 3.2. Mode of Action Studies on Host Defense Peptides

The molecular mode of action (MoA) of antibacterials is often manifold and complex and can therefore not be simply analysed using a single technique. In general, the classical microbiological assays to detect the bacterial killing kinetics (MIC values) are the essential prerequisite and selection criterion for further detailed studies. Biosensors can contribute to the elucidation of antibacterial activities, *i.e.*, when postulations on target structures exist. Biosensors are most likely promising tools to validate the binding or interaction of antibacterials with bacterial components, such as membrane constituents, to participate in the target validation process. Some of those studies will be introduced in the following section. Livne *et al*. [52] characterized OAK sequences (oligo-acyl-lysyl) of host defense peptides and identified a promising candidate (C_12_K-3β_10_). This peptide in general showed broad-spectrum activity against bacteria with activity twice as high against Gram-positive bacteria (*S. aureus*) compared to selected Gram-negative bacteria. In order to ascertain, whether these differences in the MIC are related to different peptide-membrane interactions, model membranes were used to mimic the characteristics of Gram-positives by a phosphatidylglycerol (PG):phosphatidylethanolamine (PE) mixture or of Gram-negatives by an PG:cardiolipin (CL) mixture. SPR was applied to determine the binding kinetics of C_12_K-3β_10_ to both membranes. The OAK was shown to have a higher affinity to the PG:PE membrane. Livne *et al.* proposed a deeper disruption of the membranes through the higher affinity, which would explain the lower MIC values of C_12_K-3β_10_ against *S. aureus*.

SPR was also used in elucidating the mode of action of oyster defensins. Defensins belong to the group of antimicrobial peptides (AMPs). Due to their cationic and hydrophobic nature, AMPs are thought to act by permeabilization of the bacterial membrane in a target independent way. However, Schmitt *et al.* [53] showed oyster defensins (Cg-Defm, Cg-Defh1 and Cg-Defh2) to use the cell wall precursor lipid II as defined target structure for their activity. Beside different *in vivo* and *in vitro* assays (amongst others lipid II precursor accumulation assays), SPR was applied to determine the defensins’ binding affinity to target free DOPC vesicles compared to that of DOPC/lipid II. Whereas nearly no binding of the oyster defensins was observed on DOPC vesicles, all of them significantly bound to DOPC/lipid II vesicles, thus confirming the essential role of the cell wall precursor in the oyster defensing mode of action.

### 3.3. Mass Sensitive Biosensors as Contribution to Mode of Action Studies

Mass sensitive sensors, such as quartz crystal microbalance (QCM) or the closely related surface acoustic wave sensor (SAW) have been applied in a number of studies in combination with model membrane approaches. Both techniques are based on the relationship between mass increase on a quartz crystal and a decrease in quartz crystals’ oscillation frequency. This enables the detection of the intensity and mode of membrane interaction of different peptide antibacterials and thus simulates their postulated activities at bacterial membranes.

Lipopeptides, like friulimicin, are anionic charged molecules with amphipathic characteristics and show distinct activities against Gram-positives. The leading substance daptomycin is mainly thought to interact with and disrupt bacterial membranes. Due to its net negative charge and in contrast to the AMPs discussed earlier this has to be mediated by divalent cationics like Ca^2+^. However, the structural related friulimicin was shown to not only interfere with the bacterial membrane but also to inhibit cell wall biosynthesis by complexation of the bactoprenol phosphate carrier C_55_-P in a calcium dependent manner [54]. Although the calcium-dependency of friulimicin was known, the role of calcium ions remained to be clarified.

QCM studies on friulimicin binding to model membranes not only confirmed C_55_-P as a target structure but also identified the phosphate of C_55_-P as an essential moiety for friulimicin binding. Calcium was shown to act as a bridge between the peptide and the phosphate and was further shown to affect the peptide-peptide interaction before binding to the membrane. Yet, the ability of QCM to analyze the peptide-peptide interaction in the presence of Ca^2+^ was limited. Further techniques (atomic force microscopy and circular dichroism spectroscopy) were necessary to show that calcium influences the conformation of friulimicin thus enabling the peptide to bind to the membrane [55].

However, QCM and SAW are highly suitable for the determination of kinetic constants of antibacterial binding to model membranes and thus target validation. The determination of association and dissociation rates as well as the overall binding constant KD can help to elucidate the target mediated as well as nonspecific mechanisms in detail that both contribute to the antibiotic mode of action.

The binding constants of lantibiotics were the focus of several studies. Lantibiotics are amphiphilic peptides with intramolecular ring structures that inhibit cell wall biosynthesis by binding lipid II. This mode of action is partly combined with pore forming properties. The use of QCM analysis revealed the lantibiotic nisin to have a much higher binding affinity to model membranes in the presence of lipid II compared to plain membranes (KD 0.27 μM *vs.* 1.03 μM) mainly due to a much stronger association of nisin (k_ass_ 4,677 M^−1^ s ^−1^ *vs.* 752 M^−1^ s^−1^). The combination with CV measurements illustrated the pore forming activities of nisin in dependence of lipid II [56].

**Figure 5.**
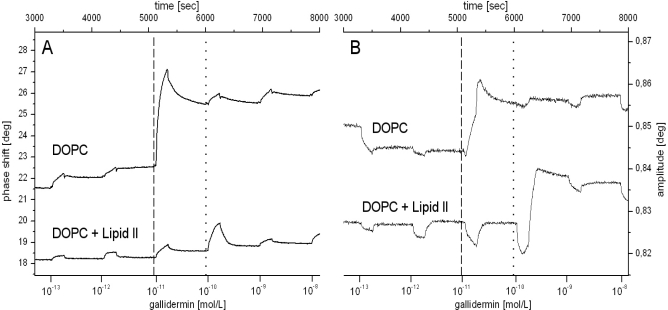
Illustration of SAW measurements. The changes in phase (**A**) and amplitude (**B**) of surface acoustic waves confirmed the lipid II recognition (dotted line in (A)) in parallel to gallidermin membrane insertion and a resulting membrane rigidification (increase in amplitude in (B) at the dashed or dotted line, resp.) (reprinted with permission from Al-Kaddah, S.; Reder-Christ, K.; Klocek, G.; Wiedemann, I.; Brunschweiger, M.; Bendas, G. Analysis of membrane interactions of antibiotic peptides using ITC and biosensor measurements. *Biophys. Chem.* **2010**, *152*, 145–152. Copyright © 2010, with permission from Elsevier).

The lantibiotic gallidermin was shown to interact with lipid II containing membranes (KD 0.28 μM) with activity comparable to that of nisin. Furthermore, gallidermin interacts with a similar intensity with lipid II free membranes (KD 0.27 μM), which contributes to the antibiotic activity in dependence on the membrane composition [57]. The detailed analysis of SAW measurements, illustrating the changes in phase and amplitude of surface acoustic waves, confirmed the lipid II recognition in parallel to gallidermin membrane insertion and resulting membrane rigidification (Figure 5).

**Figure 6.**
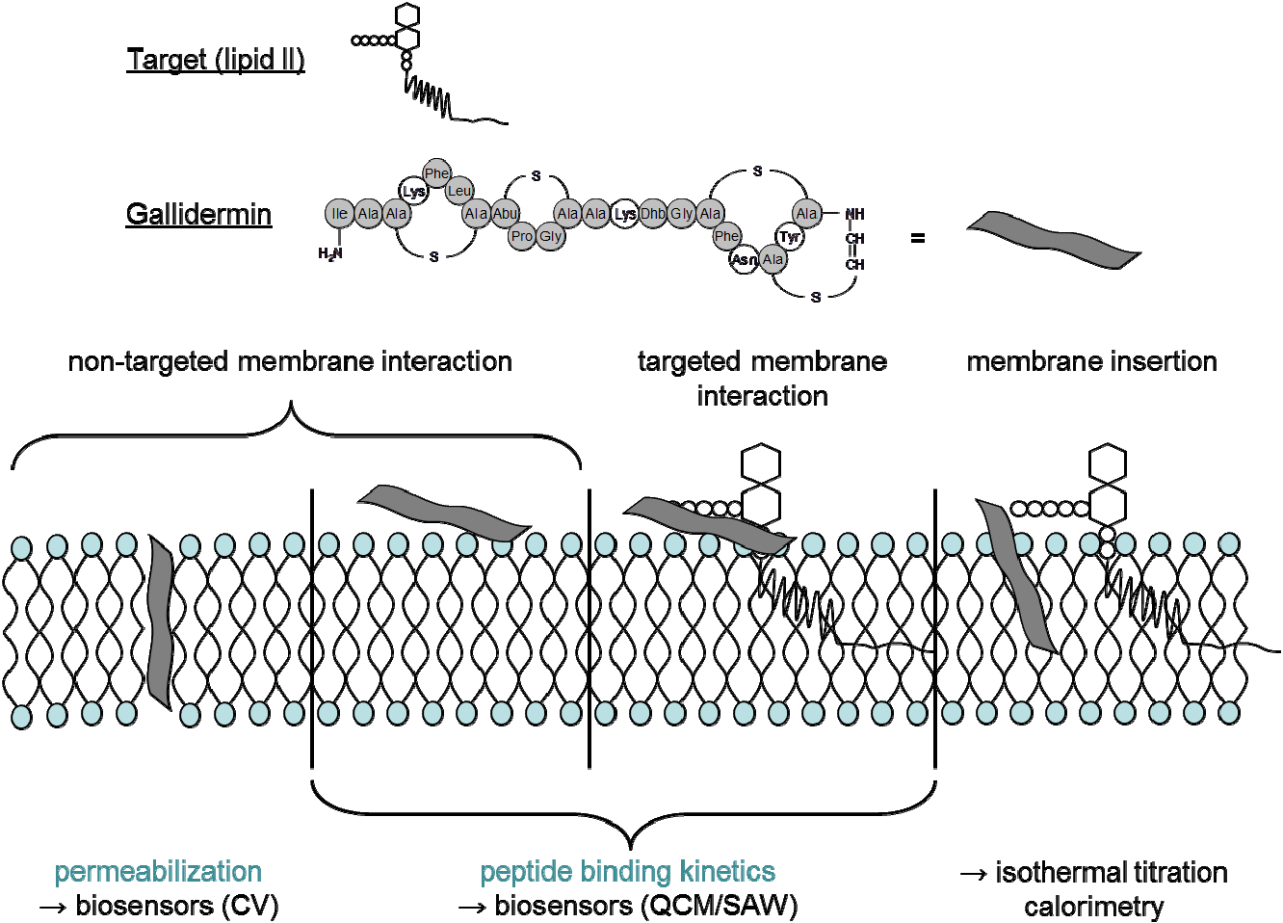
Biosensors’ contribution to the mode of action studies of gallidermin. Whereas the biosensor techniques QCM and SAW allow for the detection of binding kinetics (targeted and untargeted), additional techniques like ITC were necessary to provide evidence for non-targeted membrane activities of gallidermin in synergy with lipid II mediated interactions (reprinted with permission and minor modifications from Al-Kaddah, S.; Reder-Christ, K.; Klocek, G.; Wiedemann, I.; Brunschweiger, M.; Bendas, G. Analysis of membrane interactions of antibiotic peptides using ITC and biosensor measurements. *Biophys. Chem.* **2010**, *152*, 145–152. Copyright © 2010, with permission from Elsevier).

This illustration helped to interpret the biological MIC data. However, the mass sensitive sensors alone were not sufficient enough to fully elucidate the target independent interaction with model membranes. Therefore, SAW was combined with isothermal titration calorimetry (ITC) to finally reveal that the insertion of gallidermin into the membrane and the resultant influence on the dynamic membrane properties is a serious contribution to the gallidermin activity [58]. The above mentioned studies illustrate that biosensors are useful tools to elucidate the antibiotic mode of action of different compounds. However, due to the complexity of biological mechanisms, biosensors can only contribute to the elucidation of antibiotic activities, most likely by simulating membrane processes, but cannot be used alone for mode of action studies. For example, Figure 6 illustrates a useful combination of biosensor techniques with other analytical methods in case of gallidermin membrane interaction.

## 4. Conclusions

The present review focused on the use of biosensors to mitigate the spread of bacterial resistance. Since about two thirds of biosensor applications in the antibacterial field contribute to the detection of antibiotics in the environment or foodstuffs, this aspect has been especially emphasized. Only a limited number of recognition and detection principles have been used in this field. However, biosensors were found to be comparable to conventional methods with respect to sensitivity and specificity of antibacterial detection and thus fulfill international regulatory requirements. Since biosensors offer fast, simple and cost efficient methods that can even be used without additional sample preparation, they offer evident advantages compared to conventional analytical techniques and will therefore hold great promises for a much wider application in the near future.

In addition, a number of studies suggest that biosensors could add to the complex research to elucidate the mode of action of new antibacterial substances. However, biosensor applications for mode of action studies are presently limited to SPR and mass sensitive techniques (QCM, SAW) and dominated by the detection of kinetic binding constants of drugs or drug candidates towards their postulated targets or simulated bacterial membranes. Further studies have to justify whether biosensors will have a stronger impact on the evaluation of molecular mechanisms of antibacterial compounds.
